# Management and outcome of pregnancies in women with red cell isoimmunization: a 15-year observational study from a tertiary care university hospital

**DOI:** 10.1186/s12884-019-2525-y

**Published:** 2019-10-15

**Authors:** María Ángeles Sánchez-Durán, María Teresa Higueras, Cecilia Halajdian-Madrid, Mayte Avilés García, Andrea Bernabeu-García, Nerea Maiz, Nuria Nogués, Elena Carreras

**Affiliations:** 1 Maternal Fetal Medicine Unit, Hospital Universitari Vall d’Hebron, Ps. Vall d’Hebron 119-129, 08035 Barcelona, Spain; 2 Banc de Sang i Teixits de Catalunya, Barcelona, Spain

**Keywords:** Isoimmunization, Newborn hemolytic disease, Intrauterine transfusion, Genotype, Fetal RhD

## Abstract

**Background:**

The aims of this study were to determine the prevalence of the different anti-erythrocytic alloantibodies, to describe pregnancy outcomes according to a low-risk and high-risk classification for fetal anemia and to determine the factors that influence adverse perinatal outcomes.

**Methods:**

This retrospective observational study included women referred to our center following the identification of maternal anti-erythrocytic alloantibodies between 2002 and 2017. Pregnancies were classified as high risk for fetal anemia in cases with clinically significant antibodies, no fetal-maternal compatibility and titers ≥1:16 or any titration in cases of Kell system incompatibility. In high-risk pregnancies, maternal antibody titration and the fetal middle cerebral artery peak systolic velocity (MCA-PSV) were monitored. Low-risk pregnancies underwent routine pregnancy follow-up.

**Results:**

Maternal antibodies were found in 337 pregnancies, and 259 (76.9%) of these antibodies were clinically significant. The most frequent antibodies were anti-D (53%) and anti-K (19%). One hundred forty-three pregnancies were classified as low risk for fetal anemia, 65 (25%) cases were classified as no fetal-maternal incompatibility, 78 had clinically nonsignificant antibodies, 4 (2.8%) resulted in first-trimester pregnancy loss, and 139 (97.2%) resulted in livebirths. Of the 194 high-risk pregnancies, 38 had titers < 1:16 (resulting in 38 livebirths), and 156 had titers ≥1:16 or anti-K antibodies. In the last group, 6 cases miscarried before 18 weeks, 93 had a MCA-PSV < 1.5 multiples of the median (MoM), resulting in 3 perinatal deaths that were unrelated to fetal anemia, one termination and 89 livebirths; and 57 had a MCA-PSV > 1.5 MoM, resulting in 3 intrauterine deaths, 6 terminations and 48 livebirths. Ninety-two intrauterine transfusions were performed in 45 fetuses (87% anti-D). Adverse outcomes were related to a MCA-PSV > 1.5 MoM (*p* < 0.001), hydrops (*p* < 0.001) and early gestational age at first transfusion (*p* = 0.029)

**Conclusion:**

Anti-D remains the most common antibody in fetuses requiring intrauterine transfusion. A low or high-risk classification for fetal anemia based on the type of antibody, paternal phenotype and fetal antigen allows follow-up of the pregnancy accordingly, with good perinatal outcomes in the low-risk group. In the high-risk group, adverse perinatal outcomes are related to high MCA-PSV, hydrops and early gestational age at first transfusion.

## Background

Hemolytic disease of the fetus and newborn is a rare but life-threatening disease. Transplacental passage of maternal antibodies that bind to fetal erythrocyte antigens of paternal origin lead to fetal or neonatal hemolysis. If untreated, fetal anemia can lead to fetal heart failure, hydrops or even death.

Although anti-D alloimmunization is the most common cause of hemolytic disease of the fetus and newborn, more than 50 anti-erythrocytic alloantibodies are involved [1, 2]. The importance and proportion of non-anti-D antibodies increased [3] after the implementation of pre- and postnatal anti-D prophylaxis.

Doppler ultrasound for peak systolic velocity (PSV) measurement at the middle cerebral artery (MCA) allows for better management of the disease, avoiding invasive procedures for diagnosis of fetal anemia [4–6]. MCA-PSV measurements can detect all cases of fetal anemia, with a false positive rate of 12% [7, 8].

On the other hand, the introduction of a diagnostic test for the fetal genotype in amniotic fluid [9] and in maternal blood for certain antigens [10–12] allows the risk of fetal anemia to be ruled out in a nonnegligible percentage of cases, and, therefore, alleviates the need for thorough ultrasound-based follow-up.

The aims of this study are as follows: first, to determine the prevalence of different anti-erythrocytic alloantibodies in our center and in pregnancies that required an intrauterine transfusion; second, to describe the pregnancy outcome according to a low-risk or high-risk classification for fetal anemia; and third, to determine the factors that influence adverse perinatal outcomes.

## Methods

This is a retrospective observational cohort study including women referred to our center following the finding of maternal antibodies against fetal red blood cell (RBC) antigens between 2002 and 2017. Exclusion criteria were preconceptional counseling, positive result following anti-D administration and twin pregnancies. All data were recorded in an electronic database.

### Clinical protocol

In Rh D-negative women, prophylaxis included 300 μg (1500 IU) anti-D immunoglobulin administration at 28 weeks, after delivery if the neonate was Rh D-positive and following any of these circumstances: an invasive test, vaginal bleeding, external cephalic version, abdominal trauma, miscarriage, ectopic pregnancy or termination of pregnancy.

Since 2008, for primary prevention of alloimmunization in females of child-bearing age in our clinical practice, only Rh (D, C, c, E, e) and K compatible RBC units have been transfused in fertile women.

The prevalence of Rh D-negative in our Maternal-Fetal Medicine Unit is 15%, and the prevalence of Kell positivity is 8% (according to our blood bank and transfusion service).

Antenatal antibody screening was performed during the first trimester of pregnancy in all pregnant women and between 24 and 28 weeks only in Rh D-negative women (prior to anti-D immunoglobulin administration). Red blood cell antibody screening and identification was performed with the indirect antiglobulin test (IAT) method in Gel Cards (Anti-human globulin DG Gel®, Grifols, Spain). Antibody titration was determined by preparing serial doubling dilutions of plasma and testing them by IAT using reagent red cells showing heterozygous expression of the corresponding antigen(s). If the IAT was positive, the titration was repeated every 4 weeks or every 2 weeks if a rapid increase was detected and if the titer was close to 1:16.

**Fig. 1:**
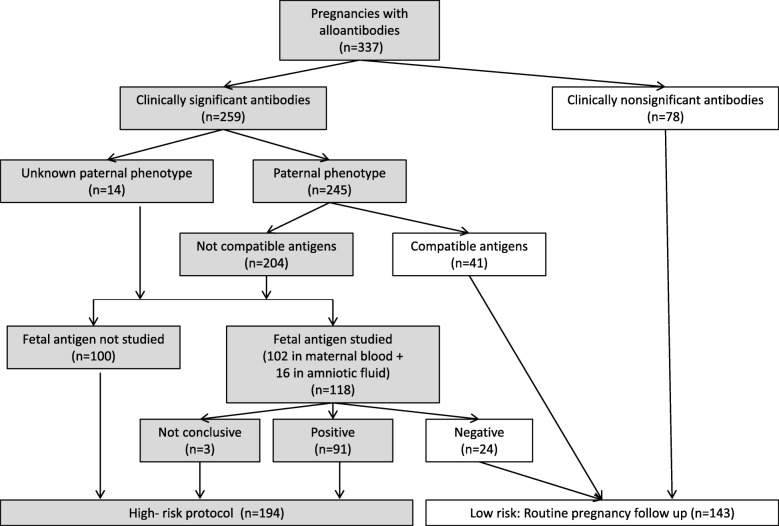
Clinical protocol

Women were referred to our center following the finding of maternal antibodies against fetal red blood cell antigens. The type of antibody was identified, and the titer was determined. For clinically significant antigens, first, the paternal erythrocyte phenotype was determined. In the case of anti-D, anti-c and anti-K antibodies, if the father had a positive phenotype and was heterozygous, the fetal genotype was determined in amniotic fluid or maternal blood, depending on the period of the study. The technique for fetal antigen-D genotyping in maternal blood has been performed in our center since 2004 and that for antigen c and Kell has been performed since 2007 (Fig. 1). The fetal RhD genotype was performed after 10 weeks of pregnancy, the Rhc genotype was performed after 16–18 weeks, and the Kell genotype was performed after 20 weeks of pregnancy. Fetal RhD genotyping was performed from cell-free fetal DNA in maternal plasma by real-time quantitative PCR with TaqMan probes for the detection of RhD exons 5 and 10. Fetal Rhc or Kell genotyping was also carried out by real-time quantitative PCR with allele-specific primers. All antibodies belonging to the Rh system, Kell system, Duffy system Fya and MNS system S antigen were considered clinically significant antibodies.

Cases with fetal-maternal blood compatibility underwent routine pregnancy care. The rest of the pregnant women with unknown or fetal-maternal incompatibility, with clinically significant antibodies, titers higher than 1:16 or any titration in the case of Kell system incompatibility, were considered high risk for fetal anemia. In these cases, weekly follow-up by ultrasound was performed, assessing the MCA-PSV, amniotic fluid volume and fetal signs of anemia or hydrops. No further IAT was performed in these cases.

When the MCA-PSV was above 1.5 multiples of the median (MoM) in two measurements taken 24 h apart, intrauterine fetal transfusion was performed if less than 34 weeks, and the baby was delivered at or after 34 weeks.

The transfusion procedure was always performed under fetal immobilization with rocuronium or vecuronium. The preferred vascular access site was the umbilical vein in the portion of the umbilical cord near its insertion into the placenta and, in exceptional cases, in the intrahepatic umbilical vein. Prior to the transfusion, a fetal blood sample was obtained to measure the fetal hematocrit, which allowed us to calculate the number of RBCs to be transfused. The blood was adequately prepared as follows: type 0 Rh CDE negative, except in anti-c cases, where the blood was Rh DCe; compatible with maternal antibodies; negative for cytomegalovirus antibodies; relatively fresh (< 7 days of age); subjected to leukodepletion by filtration; irradiated; suspended in AB plasma; and tightly packed to a final hematocrit of 75 to 85%. A second sample was obtained at the end of the transfusion to measure the final hematocrit. MCA-PSV was also used for the follow-up of the transfused fetuses. The need and timing of the second and subsequent transfusions was based on the values of MCA-PSV and the expected decline in the fetal hematocrit, according to the number of previous transfusions.

In transfused pregnancies, delivery was planned between 35 and 37 weeks depending on the intrauterine transfusion interval and last transfusion. In pregnancies where MCA-PSV was above 1.5 MoM beyond 34 weeks the baby was delivered. In pregnancies at high risk for anemia with normal MCA-PSV, delivery was planned between 37 and 39 weeks.

Birth data and neonatal follow-up were recorded, including postnatal treatment (phototherapy, immune globulin therapy, RBC transfusion or neonatal exchange transfusion) and additional complications. The indication for phototherapy was determined according to the total serum bilirubin values at the specific hourly age of the patient, gestational age at birth, and the presence or absence of risk factors for hyperbilirubinemia. Immunoglobulin therapy was indicated when the total serum bilirubin level was rising despite intensive phototherapy but did not meet the criteria for exchange transfusion. Exchange transfusion was indicated in two circumstances: a) high total serum bilirubin values, using a threshold specific to the hourly age of the patient, gestational age at birth and the presence or absence of risk factors for hyperbilirubinemia; and b) a rise in the total serum bilirubin level greater than 0.5 mg/dL per hour, despite intensive phototherapy. RBC transfusion was indicated in cases of hypovolemic shock or in cases where hyperbilirubinemia and anemia were moderate.

### Statistical analysis

All data were collected and entered anonymously into an electronic database for further analysis.

For descriptive statistics, categorical data are reported as frequencies and percentages and continuous variables are described as medians and ranges.

Univariate logistic regression analysis was performed to determine the factors related to adverse perinatal outcomes.

## Results

Four hundred and one women were referred to our center following the finding of maternal antibodies against fetal red blood cell antigens. Sixty-four women were excluded from the study, 19 had been referred for preconceptional assessment, 38 were false-positive cases or had antibodies following the administration of anti-D prophylaxis, and 7 were twin pregnancies cases.

The presence of maternal antibodies was confirmed in 337 pregnancies. Thirty-nine women were followed up in more than one pregnancy (range: 2 to 4). In 259 (76.9%) pregnancies, clinically significant antibodies were found. The distribution of antibodies is shown in Table 1. Anti-D antibody was the most frequent clinically significant antibody. Within the non-anti-D isolated antibodies, the most frequent was anti-K followed by anti-E and anti-c (Table 2). Multiple clinically significant antibodies were found in 65 (19.3%) pregnancies.

**Table 1:** Prevalence and distribution of anti-erythrocyte antibodies

Anti-erythrocyte antibodies	Frequency (*n* = 337)	(%)
Antibodies not leading to HDFN^a^	29	8.6
Antibodies rarely leading to HDFN^b^	48	14.2
Clinically significant antibodies:	259	76.9
• Anti D	138	
• Non anti-D	121	
Unidentified antibody	1	0.3

^a^anti-Lewis (*n* = 26), anti Chido (*n* = 1), anti-Jra (*n* = 1), anti P1 (*n* = 1)

^b^anti-M (*n* = 28), ABO (*n* = 9), antibodies (*n* = 3), y anti-Jka (*n* = 4), anti Vel (*n* = 2), anti- Lua (*n* = 2)

*HDFN* hemolytic disease of the fetus and newborn

**Table 2:** Distribution of clinically significant antibodies

Antibody specificities	Frequency	Percentage
Anti-D	138	53.3%
• D	90	
• D + C	31	
• D + E	2	
• D + C + E	9	
• D + G	1	
• D + G + E	1	
• D + other systems	4	
Anti-K	49	18.9%
• K	47	
• K + Rh (E)	2	
Anti-c	20	7.7%
• c	17	
• c + E	2	
• c + K + Fya	1	
Anti-E	27	10.4%
• E	22	
• E + c	4	
• E + s	1	
Anti-C	7	2.7%
• C	4	
• C + e	2	
• C + Lea	1	
Anti-e	5	1.9%
• e	3	
• e + C	1	
• e + S	1	
Anti Cw	1	0.4%
Anti Fya	2	0.8%
Anti S	10	3.9%
Total	259	100

In the group of women with clinically significant antibodies, 65 (25.1%) had paternal compatibility or negative fetal antigen and were classified as low risk (Fig. 1).

### Pregnancy outcomes (Table 3)

From the 143 pregnancies with low risk for fetal anemia, 3 (2.1%) had a first-trimester miscarriage, 1 (0.7%) opted for a termination of pregnancy at 14 weeks and 139 (97.2%) resulted in live births. One case with anti-S and autoantibodies (IgG) developed hydrops at 18 weeks and required a single transfusion.

**Table 3:** Pregnancy outcomes according to the group of risk

	Clinically nonsignificant antibodies (*n* = 78)	Clinically significant antibodies (*n* = 259)			
		Paternal compatibility or negative fetal antigen (*n* = 65)	Not compatible, fetal unknown or positive fetal antigen (*n* = 194)		
			Titer< 1:16 (*n* = 38)	Titer> 1:16 or anti-Kell (*n* = 156)	
	Low risk		High risk		
Miscarriage or TOP < 18 weeks	2 (2.6%)	2 (3.1%)	0	6 (3.8%)	
				Normal MCA-PSV (*n* = 93)	Abnormal MCA-PSV (*n* = 57)
Termination of pregnancy ≥18 weeks	0	0	0	1 (1.1%)	6 (10.5%)
Perinatal death	0	0	0	3 (3.2%)	3 (5.3%)
Livebirth	76 (97.4%)	63 (96.9%)	38(100%)	89 (95.7%)	48 (84.2%)
Intrauterine transfusion	0	1 (1.5%)	0	0	44 (77.2%)
Gestational age at birth	39.0 (27.0–42.0)	39.0 (33.0–41.0)	38.0 (26–41)	37.2 (26–42)	34.0 (27–38)

Categorical data are shown as the frequency (percentage), and continuous data are shown as the median (range)

*MCA-PSV* Middle cerebral artery peak systolic velocity

From the 194 pregnancies at high risk for fetal anemia, 6 (3.1%) resulted in a miscarriage or termination of pregnancy prior to 18 weeks, before the MCA-PSV could be measured, although none of the cases had ultrasound signs of fetal anemia.. Seven women (3.6%) opted for a termination of pregnancy after 18 weeks; one case had trisomy 21 without signs of fetal anemia, and six cases had high MCA-PSV with one due to hepatitis C infection and five due to fetal brain lesions. Of these five, one woman was referred at 26 weeks with fetal hydrops and brain lesions (hypoxic-ischemic lesions confirmed by magnetic resonance imaging) and decided to terminate the pregnancy without receiving any intrauterine transfusion. The other four had received early RBC transfusions at 19, 19, 21 and 24 weeks, and showed brain lesions on ultrasound shortly after (four cases of brain hemorrhage).

Five cases of intrauterine deaths occurred; three had undergone an intrauterine transfusion, and two had not. Neither of these two fetuses had shown previous signs of anemia; one case showed placental abruption at 30 weeks, and the other case, with a fetus without previous intrauterine transfusion, .showed chorioamnionitis at 38 weeks. The other three cases were posttransfusional deaths at 18, 22 and 26 weeks. One neonatal death occurred during labor, after a difficult breach delivery, and without previous signs of fetal anemia (Table 3).

### Intrauterine transfusions

In 58 fetuses, MCA-PSV was above 1.5 MoM. Thirteen of these cases did not receive a blood transfusion. In 11 cases that were more than 34 weeks’ of gestational age, the babies were delivered, and in two cases, the women opted for a termination of pregnancy (one with an active hepatitis C infection and the other with brain hypoxic-ischemic lesions).

**Fig. 2:**
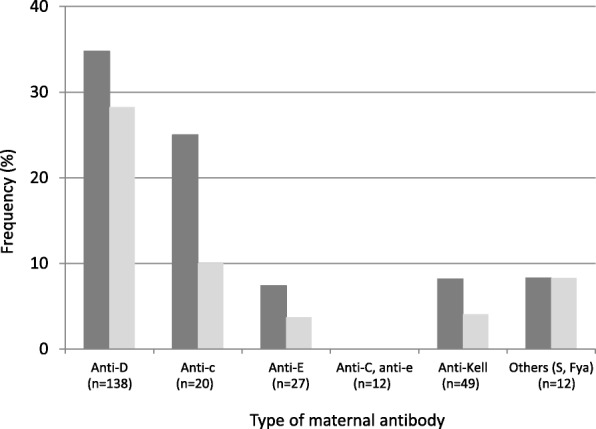
Abnormal peak systolic velocity at the middle cerebral artery (dark gray) and transfusions (clear gray) in pregnancies with clinically significant antibodies

Forty-five fetuses (13.4% of all fetuses and 23.2% of those that were high risk for fetal anemia) received an RBC transfusion, 39 fetuses (86.7%) had anti-D antibodies, 2 fetuses (4.4%) had anti-c antibodies, 2 fetuses (4.4%) had anti-Kell antibodies, 1 fetus (2.2%) had anti-E antibodies, and 1 fetus (2.2%) had anti-S antibodies. The need for a transfusion was highest in the group with anti-D antibodies (28.3%), followed by the groups with anti-c (10%) and anti-Kell antibodies (4.1%) (Fig. 2).

In one case, classified as low risk, the mother had both anti-S antibodies and autoantibodies (IgG), and the father was negative for S antigen. The fetus showed signs of anemia (hydrops and MCA-PSV > 1.5 MoM) at 18 weeks, requiring a single transfusion (pretransfusional hemoglobin 7.0 g/dL), and was delivered at 33 weeks with a postnatal hemoglobin level of 19 g/dL.

Ninety-two intrauterine RBC transfusions were performed in 45 fetuses. Twenty cases (44.4%) received a single transfusion, 13 (28.9%) cases required two transfusions, five (11.1%) cases required three transfusions, four (8.9%) cases required four transfusions, and three (6.7%) cases required five transfusions. All fetuses that required three or more transfusions had their first transfusion before 24 weeks and had anti-D antibodies (Table 4).

**Table 4:** Pregnancy outcome according to gestational age at first transfusion

Gestational age at first transfusion	n	Perinatal death	TOP	Livebirths	Number of intrauterine transfusions
< 20 weeks	6	1 (16.7%)	2 (33.3%)	3 (50%)	2.5 (1–4)
21–24 weeks	8	1 (12.5%)	2 (25.0%)	5 (62.5%)	4.5 (4–5)
25–28 weeks	15	1 (6.7%)	0	14 (93.3%)	2 (1–3)
> 28 weeks	16	0	0	16 (100%)	1 (1–3)

Categorical data are shown as the frequency (percentage), and continuous data are shown as the median (range)

*TOP* Termination of pregnancy

Overall, the posttransfusional survival rate was 96.7% (89 out of 92 transfusions). Seven fetal losses occurred among the transfused fetuses: three posttransfusional fetal deaths, all of which had fetal hydrops and were transfused at 18, 22 and 26 weeks, respectively, and four pregnancy terminations before 24 weeks due to fetal brain lesions (Table 4). Two women underwent posttransfusional emergency cesarean sections: one at 27 weeks due to sustained fetal bradycardia and the other at 34 weeks due to placental abruption. The median gestational age at delivery in this group was 34 weeks (range: 27–37), and 60.5% (23 of 38) of women underwent a cesarean section.

Nine (20%) of the 45 transfused fetuses had fetal hydrops prior to the first transfusion. Of these, 4 (44.4%) cases resulted in livebirths, 3 (33.3%) cases resulted in posttransfusional deaths, and 2 (22.2%) cases opted for termination of pregnancy. Of the 36 nonhydropic transfused fetuses, 34 (94.4%) resulted in livebirths, and 2 (5.6%) opted for a termination of pregnancy.

Adverse perinatal outcomes were significantly associated with a MCA-PSV > 1.5 MoM (OR, 9.282, 95% CI 2.99 to 28.84, *p* < 0.001), hydrops (OR 20.6, 95% CI 4.90 to 86.57, *p* < 0.001) and gestational age at first transfusion (OR 0.73, 95% CI 0.55 to 0.97, *p* = 0.029).

### Neonatal outcome

Data for neonatal outcomes were obtained for 304 of the 314 livebirths (96.8%). Neonatal treatment according to the risk group is summarized in Table 5.

**Table 5:** Neonatal treatment according to the risk group

	Clinically nonsignificant antibodies (*n* = 65)	Clinically significant antibodies			
		Compatible or negative fetal antigen (*n* = 63)	Titer < 1:16 (*n* = 38)	Titer > 1:16, Normal MCA-PSV (*n* = 90)	Titer > 1:16, Abnormal MCA-PSV (*n* = 48)
	Low risk		High risk		
Phototherapy	2/65 (3.1%)	1/63 (1.6%)	1/38 (2.6%)	53/90 (58.9%)	47/48 (97.9%)
Immunoglobulin therapy	0/64	0/62	0/38	25/90 (27.8%)	22/31 (71.0%)
Transfusion	0/64	0/63	0/38	11/90 (12.2%)	24/41 (58.5%)
Exchange transfusion	0/65	0/63	0/38	14/90 (15.6%)	27/48 (56.3%)

*MCA-PSV* Middle cerebral artery peak systolic velocity

Among the transfused fetuses, out of 38, 38 (100%) required phototherapy, 19 (50%) required immunoglobulin therapy, 21 (55.2%) required RBC transfusion, and 24 (63.2%) underwent exchange transfusion.

## Discussion

This report presents several findings. First, anti-D remains the most frequent antibody, and anti-D is associated with the highest risk for fetal anemia and a need for intrauterine transfusion, and non-anti-D antibodies account for 13% of transfused fetuses. Second, a classification of the risk of anemia as high or low based on the type of antibody, paternal phenotype, fetal antigen and antibody titration permits routine follow-up for the low-risk groups, reducing costs and maternal anxiety, with good perinatal and neonatal outcomes. On the other hand, pregnancy surveillance of high-risk groups includes intensive monitoring, with regular measurements of the MCA-PSV. Adverse outcomes in this group were related to high MCA-PSV, hydrops and early gestational age at first transfusion. Third, there was no need for neonatal RBC transfusion or exchange transfusion in low-risk pregnancies or pregnancies with titers under 1:16. In the high-risk group with titers of 1:16 or higher, a normal MCA-PSV could not rule out the presence of mild or moderate anemia that might require neonatal treatment. In our series 53% of the antibodies were anti-D, 19% were anti-K, 8% were anti-c, and 10% were anti-E. Other studies found a lower prevalence of anti-D antibodies (12.5%) and a similar prevalence of anti-K (8%) [13, 14]. The data by Awowole et al were obtained from the blood bank and transfusion service, and the data by Bollason et al. were obtained from the national birth registry, which collects data for all red cell antibodies. In our case, these figures might be biased and probably do not reflect the real prevalence in our population since women are more likely to be referred in cases of clinically significant antibodies.

The transfusion rate in our study is slightly higher than that reported by Awowole et al. (13.4% versus 7.3%), but this might also be explained by a bias in the denominator of our study, as previously mentioned. However, the distribution of antibodies among the transfused women was very similar to those of other studies in which approximately 85% of the transfused fetuses had anti-D antibodies [15–17].

The transfusion need and perinatal outcome are related to the type of antibody, with RhD isoimmunization being the most frequent and most serious during pregnancy. In our series, 46.7% of pregnancies with clinically significant antibodies were non anti-D antibodies**,** the most frequent being c, E and Kell antibodies. In addition, 13% of the transfused fetuses belonged to this group. This fact reinforces the idea of the need to perform an IAT for all pregnant women, not only for those who are RhD negative. In our study, 4.4% of the transfused fetuses belonged to the Kell system group, which was somewhat low compared to the percentage described in the literature [7].

The MCA-PSV measurement remains the method of choice to follow up pregnancies at risk of fetal anemia. It is an innocuous, inexpensive technique with a good correlation in determining the optimal time to perform intrauterine transfusion of packed RBCs. Although it loses sensitivity after 35 weeks and in poly-transfused fetuses, it is still the method used in our center, together with cardiotocographic record monitoring from 28 to 30 weeks, to assess fetal anemia. Early detection of fetal anemia, before fetal hydrops development, results in a very good survival rate. However, it is important to note that the MCA-PSV is useful to detect cases with severe anemia requiring a transfusion but does not exclude the presence of mild or moderate anemia that may require neonatal treatment.

Correct pregnancy surveillance allows optimally planning in terms of the time and place of delivery, even for pregnant women with a MCA-PSV under 1.5 MoM throughout gestation. In our series, among pregnant women with clinically significant antibodies, despite a MCA-PSV lower than 1.5 MoMs, 15.6% of newborns required postnatal exchange transfusion due to high bilirubin values. All of them had antibody titers above 1:128. Therefore, in cases of clinically significant antibodies, without maternal-fetal compatibility and with a titer > 1:16, it is always advisable to deliver at a tertiary center regardless of the MCA-PSV.

Earlier gestational age at the time of the first transfusion correlates with a greater number of transfusions. In our series, all fetuses with 3 or more transfusions had their first transfusion before 28 weeks. On the other hand, most adverse outcomes (posttransfusion death, pregnancy termination) occurred when the first transfusion was at an early gestational age; therefore, this is the group of transfused fetuses with higher risk, especially in the presence of fetal hydrops, which is why it is important to anticipate hydrops and severe anemia through proper follow-up (ultrasound and titration of antibodies in indicated cases).

Broad use of the technique of intrauterine transfusion has resulted in an overall survival rate of 96.7%, reaching 100% in nonhydropic fetuses receiving their first transfusion after 28 weeks.

The risk of fetal anemia can be ruled out by the study of the paternal phenotype. In the case of anti-Kell isoimmunization, it is especially efficient, since it results in routine gestational follow-up in 53% of the cases.

Determination of fetal antigen in amniotic fluid or maternal blood does not depend on paternity and allows strict monitoring only in gestations with a real risk of fetal anemia. In our series, 20.3% of the studied cases with a risk of fetal anemia, and with positive or unknown paternal antigen, were able to undergo routine pregnancy control.

### Clinical relevance

The findings of this study are clinically relevant in many aspects. First, the IAT should be performed for all pregnant women, regardless of their RhD antigen status, since non-anti-D antibodies can also lead to fetal anemia. Second, determination of the paternal phenotype and fetal antigen status in maternal blood or amniotic fluid permits us to rule out cases at high risk for fetal anemia, increasing the surveillance of these pregnancies and allowing routine follow up of the low risk group, with good perinatal outcomes. Third, the figures in this report provide useful information for the counseling of couples with isoimmunization.

### Strengths and limitations

One of the strengths of this study is that it was carried out in a center with a large level of experience in isoimmunization, blood transfusions and neonatal management, yielding a high number of cases and good outcomes.

One of the limitations of the study is that over the course of the lengthy study period there have been changes to the protocol, mainly the introduction of fetal antigen determination in maternal blood. Another limitation is that the data on neonatal outcomes were incomplete since those cases with low risk of fetal anemia delivered at their centers of origin.

## Conclusion

In conclusion, despite the anti-D prophylaxis policy, anti-D remains the most frequent antibody in fetuses requiring intrauterine transfusion. A classification of low or high risk for fetal anemia, based on the type of antibody, paternal phenotype and fetal antigen, allows follow-up of the pregnancy accordingly, with good perinatal outcomes in the low risk group. In the high-risk group, adverse perinatal outcomes are related to high MCA-PSV, hydrops and early gestational age at first transfusion. Neonatal treatment is only required in high-risk pregnancies with high antibody levels.

## Data Availability

The datasets used and analyzed during the current study are available from the corresponding author on reasonable request.

## Abbreviations

MCA: Middle cerebral artery
MoM: Multiple of the median
PSV: Peak systolic velocity
RBC: Red blood cell
